# Global lineages of non-tuberculous mycobacteria in residential water samples from Germany

**DOI:** 10.1186/s12866-025-04563-7

**Published:** 2025-12-07

**Authors:** Margo Diricks, Denia Frank, Inna Friesen, Stefan Niemann, Thomas A. Wichelhaus, Nils Wetzstein, the NTMscope-Eco study group, Theresa Alisch, Tobias Appel, Tobias Burbach, Gerrit Burger, Daniela Cirillo, Stephanie Faber, Sara Garcia Torres, Arash Ghodousi, Katharina Giesbrecht, Julia Hafner, Doris Heidmann, Ludwig Hofbauer, Elisabeth Holt, Raja Idris, Martin Kuhns, Lena Iliewski, Johanna Kessel, Peter Kraiczy, Sebastian Maffiold, Lina Meyer-Heinemann, Sophie Mielke, André Mohr, Martyna Olesiuk, Flavia Reyer, Sara Riedel-Christ, Christoph Stephan, Carsten Szagunn, Maria J. G. T. Vehreschild, Tobias Weirauch, Silvia Würstle, Ann-Sophie Zielbauer

**Affiliations:** 1 Molecular and Experimental Mycobacteriology, Research Center Borstel, Parkallee 1, Borstel, 23845 Germany; 2 Leibniz Research Alliance INFECTIONS, Borstel, Germany; 3 Institute of Medical Microbiology and Infection Control, Goethe University Frankfurt, University Hospital, Frankfurt am Main, Germany; 4 Supranational Reference Center for Mycobacteria, Research Center Borstel, Borstel, Germany; 5 German Centre for Infection Research (DZIF), Partner Site Hamburg-Lübeck-Borstel-Riems, Borstel, Germany; 6 EPHE, PSL University, and Institut de Systématique, Evolution, Biodiversité, ISYEB, Muséum National d’Histoire Naturelle, CNRS, Sorbonne Université, EPHE, Université des Antilles, Paris, France; 7 Department of Internal Medicine, Infectious Diseases, Goethe University Frankfurt, University Hospital, Frankfurt am Main, Germany; 8 German Center for Infection Research (DZIF), Associated Partner, Frankfurt, Germany; 9 Mycobacterial Infection Research Unit (MIRU), Goethe University Frankfurt, Frankfurt am Main, Germany

**Keywords:** NTM, Non-tuberculous mycobacteria, Environmental, *Mycobacterium chimaera*, *Mycobacterium chelonae*, *Mycobacterium abscessus*, Dominant circulating clones, Transmission

## Abstract

Non-tuberculous mycobacteria (NTM) are ubiquitous environmental organisms that can cause severe infections in vulnerable individuals. However, the environmental reservoirs and transmission routes of clinically relevant NTM remain insufficiently understood. Given the rising incidence of NTM infections, understanding where these bacteria thrive can inform public health measures and reduce infection risk. In this study, we analysed the prevalence and genetic diversity of mycobacteria in indoor water samples from private residences and a hospital in Frankfurt am Main, Germany, and characterised them using whole genome sequencing and phenotypic drug susceptibility testing. Core genome multi-locus sequence typing (cgMLST) was employed to compare environmental isolates with nearly 3,000 clinical and environmental NTM isolates from around the world, aiming to identify potential epidemiological connections and global lineages. NTM were isolated from 9 of 41 households (22.0%) and 15 of 102 water samples (14.7%). Eight different NTM-species were identified, including one novel species. For *Mycobacterium abscessus* and *Mycobacterium chimaera*, strains belonging to globally distributed lineages were detected. Notably, *M. abscessus* dominant circulating clone 3 (DCC3) was present in shower water and we report, for the first time, global lineages of *M. chelonae* in both environmental and clinical samples. While NTM from different households were genetically distinct, isolates from five households (*M. chimaera*, *M. abscessus*, and *M. chelonae*) were closely related to clinical isolates from Germany and abroad. In conclusion, our findings highlight indoor water systems as reservoirs for some clinically significant NTM, including globally relevant clones such as *M. abscessus* DCC3, and underscore the potential for targeted public health interventions to reduce exposure risks, especially for vulnerable populations.

**Supplementary Information:**

The online version contains supplementary material available at 10.1186/s12866-025-04563-7.

## Introduction

Non-tuberculous mycobacteria (NTM) comprise all mycobacteria except *Mycobacterium tuberculosis* complex (MTBC), *M. leprae* and *M. lepromatosis* [1]⁠. To date, more than 200 NTM species have been described which can be divided into rapid-growing mycobacteria (RGM, usually showing growth on solid media within 7 days) and slow-growing mycobacteria (SGM, showing growth after more than 7 days) [2]⁠.

Several NTM species are opportunistic pathogens with variable virulence, primarily affecting the lungs and causing progressive disease in susceptible individuals, particularly those with chronic lung conditions such as chronic obstructive pulmonary disease (COPD), bronchiectasis, cystic fibrosis, and prior tuberculosis [1, 3–5]⁠. Although pulmonary infections are most common, extrapulmonary disease can occur, typically presenting as cervical lymphadenitis in children, skin and soft tissue infections following inoculation, or disseminated disease in immunocompromised individuals, such as those living with HIV/AIDS [6]⁠. In countries with a decreasing incidence of tuberculosis (TB), NTM infections are considered an increasing health concern. In Europe, *M. avium* complex (MAC), *M. kansasii*, *M. abscessus* and *M. xenopi* are most commonly isolated from clinical samples of NTM patients [7]⁠.

Unlike MTBC, NTM are ubiquitous bacteria that have also been isolated from various environmental sources within and beyond the health care setting [8–10]⁠. Different NTM species appear to occupy distinct ecological niches, including aquatic environments such as showerheads, as well as dust and soil [11–13]⁠. The fact that NTM are present in a variety of environments and habitats including showers in patients’ homes raised the question, whether NTM-infections are possibly transmitted via aerosols to the patients’ lungs [14]⁠. In the USA, 81.1% of water samples from households in Philadelphia have been demonstrated to be contaminated with *M. avium* [15]⁠. On the other hand, 44% of indoor water samples in Hawaii were tested positive for NTM, with *M. avium* being rarely isolated [10]⁠. Thus, different geographical regions seem to exhibit different isolation rates as well as species distributions of NTM recovered [13, 16]⁠. In Germany, data on the prevalence of NTM isolation from environmental samples is scarce. An early study from Peters and coworkers identified mycobacteria in 50/118 (42.4%) water samples in 21/30 (70%) sites inside homes or hospitals in Berlin [17]⁠. Lahiri and coworkers found *M. avium* subsp. *hominissuis* in 33% of dust samples, 20% of soil samples but not in water samples that were collected from tap water, lakes, fountains, rivers and rain puddles [12]⁠.

*M. intracellulare* subsp. *chimaera* (further referred to as *M. chimaera)*, another MAC species, is able to build water associated biofilms and has been involved in a worldwide outbreak among patients that underwent cardiac surgery [18]⁠. Here, water tanks within heater cooler units (HCU) used during the procedures have been contaminated with a clonal *M. chimaera* outbreak strain (Zuerich-1) [19]⁠. This strain was supposed to be descended from a lineage already circulating in patients with pulmonary conditions prior to the outbreak [20]⁠.

Besides the MAC, rapid-growing NTM, such as *M. chelonae* and *M. fortuitum,* were frequently isolated from contaminated water sources and involved in different outbreaks associated with tattoo ink, cosmetic surgery, or cardiac surgery [21–23]⁠. Among the RGM, *M. abscessus* is notoriously responsible for difficult-to treat infections, especially in patients with predisposing lung conditions, such as cystic fibrosis [24]⁠. Here, multiple whole genome sequencing studies confirmed the presence of dominant-circulating clones (DCCs), which are defined as groups of closely related *M. abscessus* strains that are frequently isolated from patients across the world and are thought to be associated with higher virulence, higher resistance rates and worse clinical outcomes [25]⁠. Their transmission is supposed to take place via fomites or aerosols and only recently have they been described to be present in drinking water samples from Australia [26, 27]⁠.

The aim of this study was to assess the occurrence and diversity of NTM recovered from household water samples in a confined geographical area in Germany using whole genome sequencing, with a particular focus on determining whether the detected isolates represented globally distributed lineages or locally restricted strains.

## Methods

### Environmental water samples and bacterial culture

Water samples were retrieved from randomly chosen households and one hospital in the Metropolitan Region of Frankfurt am Main, Germany, from 10/2022 to 11/2023. We obtained 500 ml of tap water or water retrieved from shower heads (Figure S1) in 1L sterile flasks coated with 20 mg sodium thiosulfate (Engellabor, Heusenstamm, Germany). Metadata including the type of specimen, its geographical origin represented by postal codes and the sampling time point was collected with an online data collection tool (https://www.jotform.com).

After a maximum storage duration of 24 h at room temperature, samples were further processed (a majority on the same morning). To prevent growth of bacteria other than mycobacteria, 500 mL of a *0.005%* Cetylpyridinium chlorid-Monohydrate-solution (CPC) (Carl Roth, Karlsruhe, Germany) was added – resulting in a total volume of 1L – and incubated at room temperature for 30 min [28]⁠. Then each sample of 1000 ml was cumulatively filtered with an EZ-Fit manifold filtration unit at negative pressure and a single Microfil 47 mm/0,45 µm/100 ml filter (Merckmillipore, Darmstadt, Germany) to increase bacterial concentration. The filter membrane was removed from the filtration unit and rinsed with 2 ml of sterile water (Fresenius Kabi, Bad Homburg, Germany) which was collected in a Falcon tube and subsequently used for bacterial culture. Five Middlebrook agar 7H10 plates (Becton Dickinson, Heidelberg, Germany) and one chocolate agar plate (Oxoid, Wesel, Germany) were streaked with 100 µl of the resulting flushing liquid, each. Middlebrook agar plates were cultivated at 37° C for a duration of eight weeks or until visible growth was detected. Chocolate agar plates were cultivated for seven days at 37 °C as control. For all experiments a negative control with 500 ml sterile water and 500 ml CPC was performed.

### Species identification and susceptibility testing

Colonies from positive cultures were subjected to Ziehl–Neelsen staining. When acid fast bacilli (AFB) were detected, sub-cultures of these samples underwent internal transcribed spacer-(ITS)-PCR and for MAC-species the Genotype NTM-DR (Hain Lifesciences, Nehren, Germany) for initial mycobacterial species identification [29]⁠. Samples yielding positive cultures for non-acid-fast bacteria were excluded from further analyses.

All environmental NTM isolates underwent phenotypic drug susceptibility testing (pDST) using the RAPMYCO1 plate (Thermo Fisher Scientific, Waltham, USA) for RGM and the SLOMYCO1 plate (Thermo Fisher Scientific, Waltham, USA) for SGM as recommended by CLSI and the manufacturer. Breakpoints for different antibiotic agents were retrieved from CLSI [30]⁠.

### Whole genome sequencing

All isolates from indoor water samples identified as *Mycobacterium* spp. underwent whole genome sequencing (WGS) at the Research Center Borstel, Leibniz Lung Center Germany. In addition, WGS was performed on 49 M*. chelonae* isolates that were retrieved for routine diagnostics at the Research Center Borstel during 2021 and 2022 as well as 10 M*. chelonae* strains isolated at University Hospital Frankfurt. Briefly, DNA was extracted using the Cetyltrimethylammonimum bromide (CTAB) method as described previously [31]⁠**.** Next-generation sequencing libraries were generated from extracted genomic DNA with a modified Illumina Nextera library kit protocol [32]⁠. Then, libraries were sequenced in a 2 × 150-bp paired-end run on an Illumina NextSeq 2000 instrument (Illumina, San Diego, CA, USA).

### Bioinformatical analysis

Obtained raw reads were subjected to our in-house pipeline NTMseq [33]⁠, which includes quality control with FastQC v0.11.4 [34]⁠ and MultiQC v1.13.dev0 [35]⁠, contamination detection with kraken2 2.1.2 [36]⁠ using the standard database (v.2020-09-06), NTM species determination using NTM-Profiler v.040 [37]⁠, assembly with shovill v.1.1.0 using spades v.3.15.0 [38, 39]⁠, resistance prediction using NTM-Profiler v.040 and AMRfinder + v. 3.11.2 using reference gene database v.2023-09-26.1, with default values as well as relaxed detection thresholds of 50% identity and 50% coverage, and plasmid prediction using SRST2 v.0.2.0 using a reference database consisting of 208 mycobacteriaceae plasmids retrieved from PLSDB v2023-11-03 [40, 41]⁠. Draft assemblies were also submitted to the type strain genome server (TYGS) (https://tygs.dsmz.de/) for species identification and phylogenetic inference with mycobacterial reference genomes. For the latter, all pairwise comparisons among the set of genomes were conducted via the TYGS web server using Genome BLAST Distance Phylogeny approach (GBDP) and accurate intergenomic distances inferred under the algorithm ‘trimming’ and distance formula d5 [42]⁠. 100 distance replicates were calculated each. The resulting intergenomic distances were used to infer a balanced minimum evolution tree with branch support via FASTME 2.1.6.1 including SPR postprocessing [43]⁠. Branch support was inferred from 100 pseudo-bootstrap replicates each. The trees were rooted at the midpoint and visualized with PhyD3 [42, 44]⁠.

Core genome multi-locus sequence typing (cgMLST) was conducted using SeqSphere + v.9.0.10 to evaluate the genetic relatedness among NTM isolates from indoor water samples, clinical and animal samples from Germany, and publicly available samples collected worldwide (Tables S1-S3). For *M. abscessus*, a previously described cgMLST scheme comprising 2904 genes was used [25]⁠ while for *M. chelonae* and *M. chimaera* two new ad hoc cgMLST schemes were developed (*table S4*) consisting of 3461 and 3719 genes (Supplementary Table S4), respectively (Diricks et al., in preperation). Only sequences with ≥ 95% good cgMLST targets and < 6 Mbp (*M. abscessus*), ≤ 7.6 Mbp (*M. chimaera*) and < 6.2Mbp (*M. chelonae*) genome length were included. Neighbor joining and minimum spanning trees were generated in SeqSphere + based on cgMLST derived distance matrices. Trees were visualised and edited using iTOL and ggtree [45, 46]⁠.

*M. chimaera* lineages were determined based on signature SNPs previously determined [19].

### Statistical analysis

All statistical analyses have been performed in R v. 4.3.1 (“Beagle Scouts”) [47]⁠. Categorical data is summarised as frequency with percentage. The Fisher-exact test was used to test for differences in occurrence between different sample sources. Graphs and maps were drawn using the *ggplot* and *ggmap* packages, respectively [48, 49]⁠.

## Results

### Recovery of NTM and species distribution from collected samples

**Fig. 1:**
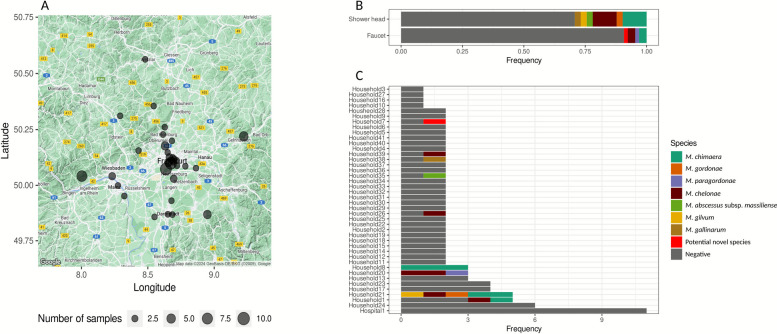
Map of sampling sites (**A**), mycobacterial species and positivity rate by sample origin (**B**), and species distribution within different households (**C**)

Overall, 102 individual water samples from 41 different households and one hospital in the Rhine-Main-Metropolitan Area were collected between 10/2022 and 11/2023 (Fig. 1A). From those, NTM were recovered in 9/41 households (22.0%) and 15/102 samples (14.7%), respectively. NTM were significantly more frequent in shower head derived water than in tap water (29.3% vs. 9.2%, OR = 4.0, *p* = 0.01) (Fig. 1B). All mycobacterial cultures from hospital water samples remained sterile (Fig. 1C*)*.

Overall, 18 isolates belonging to 8 different NTM-species were identified (Table 1). Among the RGM, *M. chelonae* was most frequent (*n* = 6) while *M. gilvum, M. gallinarum,* and *M. abscessus* subsp*. massiliense* were recovered one time each. Among the SGM, *M. chimaera* was predominant (*n* = 6) and singular isolations of *M. paragordonae*, *M. gordonae*, and one potentially novel species were observed (Fig. 1C). The potential new species was most closely related to *M. chimaera* (93% ANI). Three samples contained polymycobacterial communities comprising two different NTM species (*M. chelonae* + *M. paragordonae, M. gordonae* + *M. chimaera,* and *M. gilvum* + *M. chelonae)*. One household delivered water samples with 5 different NTM strains (*M. gilvum, M. chelonae, M. gordonae,* and two genetically different strains of *M. chimaera*) from three samples. Figure S2 shows an overall phylogeny of included isolates and comparative reference genomes.

**Table 1:** Isolated NTM from water samples, sampling sites and molecular species identification

Strain	Household	Sampling site	Runyon-Group	ITS-PCR/LPA	WGS (NTM-Profiler)
ntmscope_eco_1	Household1	Shower head	SGM	*M. chimaera*	*M. chimaera*
ntmscope_eco_2	Household1	Faucet	RGM	*M. chelonae*	*M. chelonae*
ntmscope_eco_3	Household7	Faucet	SGM	*M. chimaera* (result not unambiguous)	Novel species
ntmscope_eco_4	Household8	Shower head	SGM	*M. intracellulare* (result not unambiguous)	*M. chimaera*
ntmscope_eco_5	Household8	Faucet	SGM	*M. intracellulare* (result not unambiguous)	*M. chimaera*
ntmscope_eco_6	Household8	Shower head	SGM	*M. intracellulare* (result not unambiguous)	*M. chimaera*
ntmscope_eco_7	Household20	Faucet	SGM	*M. gordonae* (result not unambiguous)	*M. paragordonae*
ntmscope_eco_8	Household20	Faucet	RGM	*M. chelonae*	*M. chelonae*
ntmscope_eco_9*	Household20	Shower head	RGM	*M. chelonae*	*M. chelonae*
ntmscope_eco_10	Household21	Shower head	RGM	*Mycobacterium spp.*	*M. gilvum*
ntmscope_eco_11	Household21	Shower head	RGM	*M. chelonae*	*M. chelonae*
ntmscope_eco_12	Household21	Shower head	SGM	*M. chimaera*	*M. chimaera*
ntmscope_eco_13	Household21	Shower head	SGM	*M. gordonae*	*M. gordonae*
ntmscope_eco_14	Household21	Faucet	SGM	*M. chimaera*	*M. chimaera*
ntmscope_eco_15	Household26	Shower head	RGM	*M. chelonae*	*M. chelonae*
ntmscope_eco_16	Household35	Shower head	RGM	*M. abscessus* *ssp. massiliense*	*M. abscessus* subsp. massiliense
ntmscope_eco_17	Household38	Shower head	RGM	*M. gallinarum*	*M. gallinarum*
ntmscope_eco_18	Household39	Shower head	RGM	*M. chelonae*	*M. chelonae*

*ITS-PCR* internal transcribed spacer polymerase chain reaction, *LPA* line probe assay, *WGS* whole genome sequencing, *NTM* non-tuberculous mycobacteria, *SGM* slow growing mycobacteria, *RGM* rapid growing mycobacteria

^*^This isolate was contaminated with proteobacteria and excluded from subsequent analyses

### Antimicrobial susceptibility and predicted plasmid content of recovered NTM isolates

Within the environmental SGM, we did not observe any isolates being phenotypically resistant to macrolides or aminoglycosides (Tables S5 and S6). However, the majority of *M. chelonae* isolates showed intermediate susceptibility to amikacin, while all RGM were fully susceptible to macrolides. Using the default values for AMRfinder +, only one resistance gene was found in the whole dataset: blaMAB in the *M. abscessus* strain. Using more relaxed thresholds, at least one resistance gene was detected in all strains except for the potential novel species (Figure S3).

Representatives of 10 plasmid clusters were detected in 7 environmental NTM isolates (all *M. chimaera* or the closely related potential novel species), among those, plasmids 1, 2 and 5 from the Zuerich-1 Strain and plasmid 4 from the Zuerich-2 strain were found (Figure S4).

### Phylogenetic relations of environmental *M. chimaera* isolates and association with globally distributed lineages

**Fig. 2:**
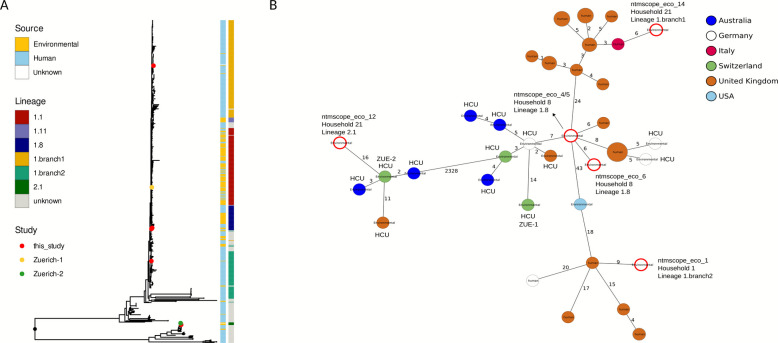
Phylogeny of 6 *M. chimaera* isolates recovered from environmental samples in this study (red dots) and 607 publicly available sequences from human and environmental samples (**A**) and minimum spanning tree of six environmental *M. chimaera* isolates (highlighted with red-bordered circle) from Frankfurt together with closely related isolates (≤ 10 alleles) and Zuerich-1 (ZUE-1) based on 3719 cgMLST targets (**B**) HCU: heater cooler unit

In total, six *M. chimaera* strains belonging to 4 previously described lineages (1.8, 2.1, 1.branch1 and 1.branch2) were isolated from three different households. These were set into a global context with international environmental and clinical isolates (*n* = 613 total) (Fig. 2, Figure S5). Water samples from household 8 yielded three lineage 1.8 M*. chimaera* isolates (Table 1). While the strains from the tap water and shower head in the main bathroom were identical (0 alleles difference), a second strain from the shower of a guest bathroom was genetically distinct but closely related to these (6 alleles difference). Further, these isolates showed a minimum distance of 14 alleles with the HCU-related outbreak strain Zuerich-1 (lineage 1.1) while the median allelic distance between HCU water reservoir isolates (including Zuerich-1) and cardiac surgery-related patient isolates was 8 alleles. In contrast to household 8, the two *M. chimaera* isolates from household 21 belonged to different phylogenetic groups (lineage 2.1 and group 1 1.branch1). None of the *M. chimaera* environmental isolates from this study was most closely related to the German clinical isolates (Fig. 2B).

### Phylogenetic relations of environmental *M. chelonae* isolates and identification of globally distributed lineages

**Fig. 3:**
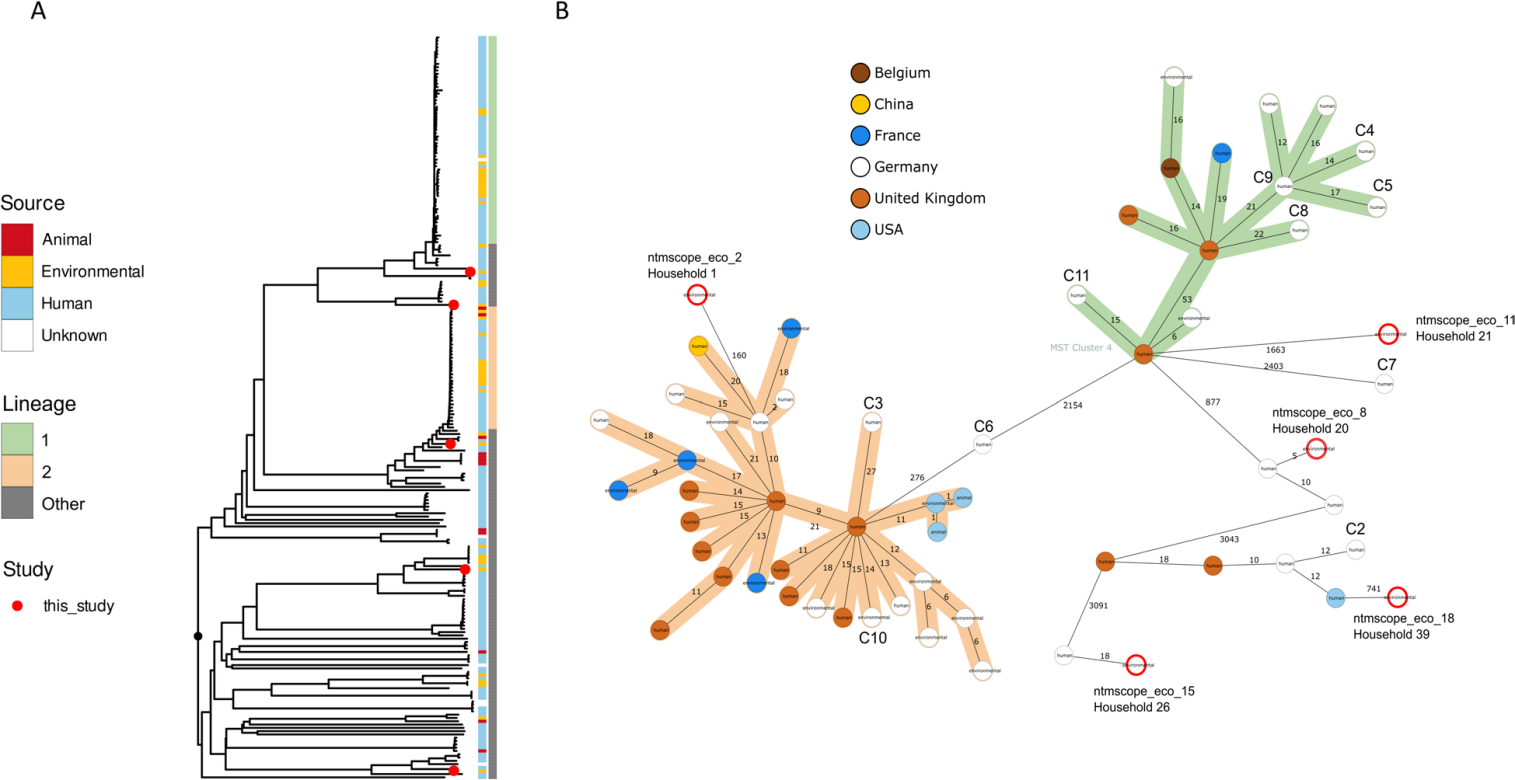
Phylogeny of 225 *M. chelonae* isolates including environmental samples isolated as part of this study in Frankfurt (red dots) (**A**) and minimum spanning tree of 5 environmental *M. chelonae* isolates from Frankfurt (highlighted with red-bordered circle) and 10 clinical isolates from Frankfurt (indicated as C2-C11) together with closely related isolates (≤ 25 alleles) based on 3461 cgMLST targets. (**B**) The newly identified global lineages 1 (green) and 2 (orange) correspond to d50 cluster 1 and d50 cluster 2, respectively, defined using a distance threshold of 50 alleles (i.e. each member of the cluster is linked to at least one other member within a maximum distance of 50 alleles, although the pairwise distance between some members of the same cluster may exceed 50 alleles)

Five out of six *M. chelonae* isolates recovered from household water samples in Frankfurt and ten clinical isolates from patients at a Frankfurt hospital were compared with 55 additional *M. chelonae* isolates from Germany and with publicly available samples from other countries (*n* = 225 total) (Fig. 3 and Figure S6). Environmental *M. chelonae* isolates from Frankfurt were not closely related to clinical samples from Frankfurt (min. distance 162 alleles) but one was closely related to a clinical sample from another German hospital (5 alleles) (Fig. 3B). Some clinical isolates from Frankfurt belonged to the same cluster but with distances > 10 alleles. Overall, the phylogenetic tree showed two large clusters comprising isolates of human, environmental or animal origin from different countries, with most samples within the clusters connected with less than 50 alleles (Fig. 3A and Figure S6).

### Phylogenetic relations of the environmental *M. abscessus* isolate and association with dominant circulating clones

**Fig. 4:**
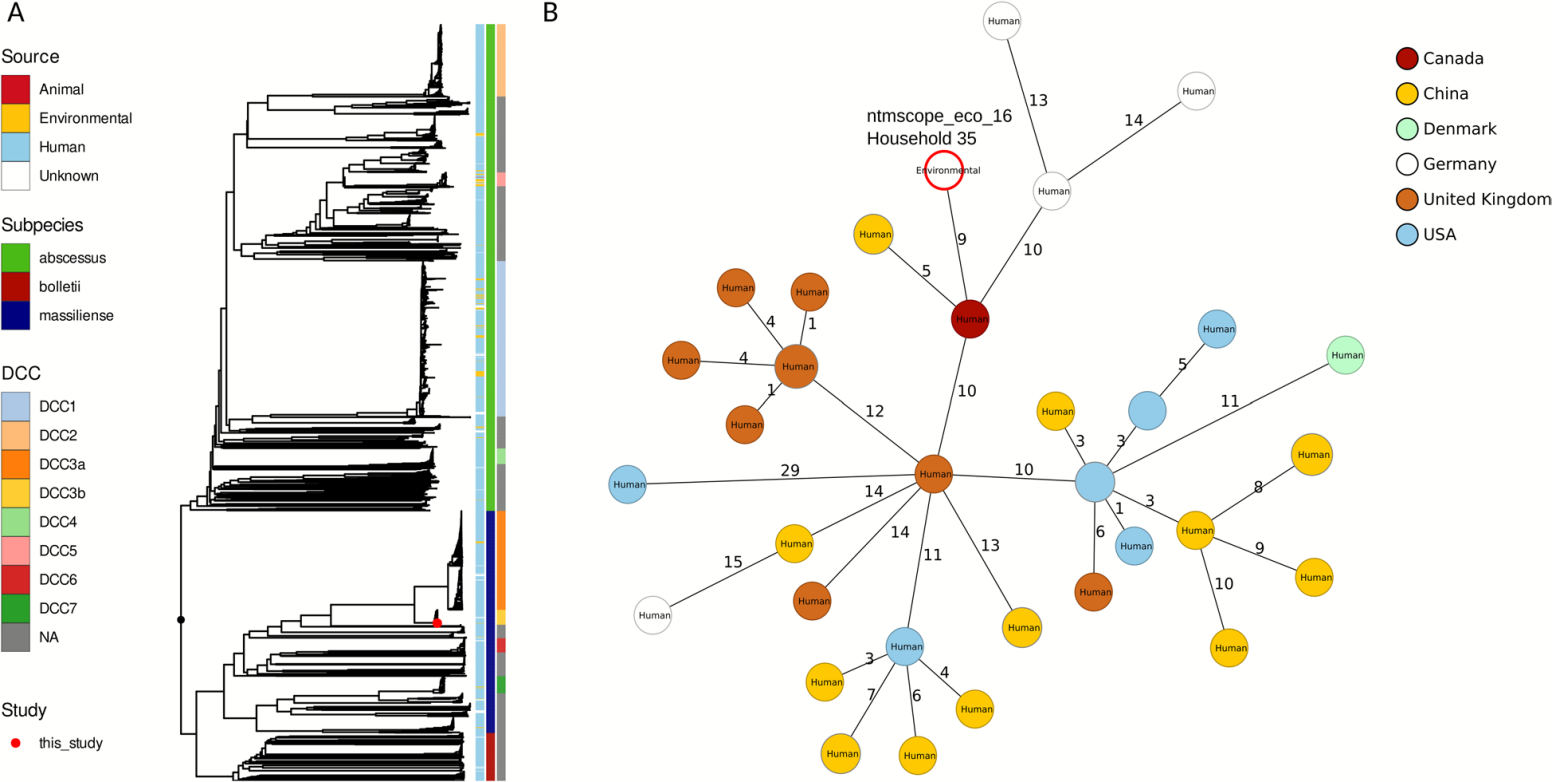
Phylogeny of one *M. abscessus* subsp. *massiliense* isolate recovered from an indoor water sample in this study (red dot) belonging to DCC3b and public sequences (**A**) and minimum spanning tree of one environmental *M. abscessus* subsp. *massiliense* isolate from Frankfurt (highlighted with red-bordered circle) as well as 41 other DCC3b strains based on 2904 cgMLST targets (**B**)

We constructed a global phylogeny of sequences from clinical and environmental *M. abscessus* isolates (*n* = 2132 total) (Fig. 4 and Figure S7). The single environmental *M. abscessus* isolate from our study (ntmscope_eco_16) belonged to ST37 and dominant circulating clonal complex DCC3b (Fig. 4A). It was most closely related to a strain isolated in Canada in 2019 (GD44, GCF-017176395.1) with a genetic distance of only 9 alleles (Fig. 4B). The closest German isolate (ERR7253760) was 15 alleles apart and isolated from a patient with cystic fibrosis at our center in 2019 [24]⁠.

Interestingly, a large majority (77%, 79/102) of environmental samples (78 public and one from our study) were shown to be DCCs, with 52 isolates belonging to DCC1, 6 to DCC3, 18 to DCC5 and 3 to DCC7. In addition, 72/102 (71%) of environmental isolates clustered with 10 or less alleles with at least one other isolate in a total of 19 different clusters consisting of 2–29 isolates. Of these 19 clusters, 11 consisted both of human and environmental isolates.

## Discussion

In this study, we provide comprehensive genomic and phenotypic data of NTM isolates from indoor water samples in Frankfurt am Main, Germany. We detected potentially clinically relevant NTM species from tap water and showerheads with *M. chimaera* and *M. chelonae* among the most frequently isolated species. Phylogenetic analysis revealed clustering of isolates from environmental samples with local and global patient isolates indicating potential transmission chains. We demonstrate the recovery of a global clone of *M. abscessus* from a household shower in Germany, as well as *M. chimaera* strains closely related but not belonging to the outbreak clone Zuerich-1. Further, we provide the first insights into the genomic population structure of *M. chelonae* indicating the presence of two globally distributed lineages encompassing isolates from animal, water and human sources.

In our study, 22.0% of households and 14.7% of water samples were found to contain detectable amounts of NTM. Previous studies have reported higher recovery rates [50–55]: e.g. 42.9% and 81.1% of indoor water samples in the US were shown to be positive for NTM, and 95% of water samples from 20 NTM patients in Australia [14, 15]⁠. Also in Berlin, Germany, higher rates of NTM detection with a predominance of *M. gordonae* were detected in the nineties [17]. However, in this study, mycobacteria were mainly recovered from hospital water samples while all of our hospital water samples remained negative. This might be linked to the fact that water filters installed at faucets have become standard in our facility. Nevertheless, these discrepancies might not only be explained by variations in water networks or water treatment and different environmental conditions (e.g. climate and elevation) in the respective countries [13, 56, 57]⁠, but also by methodological factors. Specifically, culturing exclusively on solid media incubated at 37 °C, may have contributed to lower detection rates in our study. A differential susceptibility to decontamination agents such as CPC and Na thiosulfate might also have influenced recovery, potentially skewing the species distribution toward NTM that are more resistant to these disinfectants. Further, our sampling strategy did not include a refrigeration step. However, the majority of samples was processed within the same morning and only a few have been stored overnight at room temperature. Finally, sampling in households of patients with clinically relevant NTM disease might increase pre-test probability in other studies. As we only sampled in private households of individuals without known NTM disease, and in a hospital with a filtering system, this might also explain for lower detection rates in our study.

Phenotypic drug susceptibility testing did not identify any environmental isolates with macrolide or aminoglycoside resistance. This finding might be attributed to the fact that these resistances often occur after previous antibiotic therapy in patients with relevant clinical disease [58]⁠. Further, we identified previously known plasmids in environmental *M. chimaera* isolates that have been associated with the outbreak strains Zuerich-1 and Zuerich-2 [19]⁠. Apparently, these seem to be present not only in patients and the nosocomial environment but also in homes of healthy individuals [59]⁠.

Among the MAC species, we only detected *M. chimaera* isolates in our samples, but not *M. avium* or *M. intracellulare*. This is in line with previous data from Lahiri et al. who isolated *M. avium* in Germany only from dust or soil, but not from water samples [12]⁠. A species-specific environmental habitat seems to also confirm previous data from Wallace et al.: here, MAC species from water biofilms were identified to be mainly *M. chimaera* in the US [11]⁠.

Additionally, we isolated a *M. abscessus* subsp. *massiliense* strain belonging to DCC3b (ST37) from a shower head. The presence of clonally related *M. abscessus* strains (i.e. belonging to the DCCs) in drinking water samples from Australia was recently described [27]⁠. DCCs represent the most prevalent *M. abscessus* strains found in patients worldwide [25]⁠. Thus, beyond the previously suggested theory that these strains have spread globally through intercontinental transmission, it is also possible that they are simply the most prevalent species in the environment. To the best of our knowledge, this is the first reported instance of identifying a *M. abscessus* DCC3 in an environmental sample from Europe.

Further, we detected the rapid growing NTM species *M. chelonae* in several water samples. This species is mainly involved in soft tissue and wound infections, as well as surgical site infections, e.g. after cosmetic or cardiac surgery [23]⁠. Recently, an outbreak of *M. chelonae* infections was traced to contaminated bioprosthetic heart valves, resulting in implant malfunction [23]. However, when isolated from the lungs it is rarely of clinical relevance [60]⁠. Interestingly, our global phylogeny also provided for the first time evidence of two major *M. chelonae* lineages containing closely related strains (many < 50 alleles apart) that were isolated in different countries on different continents. Both *M. chelonae* lineages were found in clinical strains but also in fish and different environmental samples including tap water, surface water, catheters and even HCUs. Their global distribution may provide an initial indication of DCCs, similar to those described in *M. abscessus*, but confirmation will require larger datasets and experimental evidence linking these lineages to specific phenotypic traits such as increased virulence, transmission, antimicrobial resistance, and/or biofilm formation. Nevertheless, our global phylogeny offers the first insights into the population structure of *M. chelonae* and serves as a valuable foundation for investigating previous and future outbreaks.

We did not observe geographical clustering of environmental isolates from different households in Frankfurt. However, the observation that several clinical and environmental isolates—some from other regions in Germany, others from abroad, including samples collected as early as 2019—differ by fewer than 10 alleles from the Frankfurt environmental isolates raises questions about possible transmission routes and points to the importance of environmental reservoirs for NTM spread. Considering previously published mutation rates of mycobacteria like *M. abscessus* and *M. tuberculosis* – around 0.2–0.5 alleles/genome/year [25, 61, 62]⁠, this indicates these patients might have been in contact with the same water distribution system of the investigated households in Frankfurt, perhaps via international travel within last decade. On the other hand, the isolates could also be part of a widely distributed environmental lineage that has persisted globally and is genetically stable at least at the core genome level. This hypothesis seems particularly plausible for *M. chimaera*, which shows a very clonal population structure for the majority of strains. These findings underscore the need to interpret genomic similarity in the context of detailed epidemiological data and to apply stringent criteria when investigating potential outbreaks.

This study has several limitations: first, no households of patients with NTM pulmonary disease or other NTM-infections were screened, which may have reduced the pre-test probability. However, we nevertheless demonstrate the presence of global lineages in water samples underlining their ubiquity. Second, the sample size and our low detection rate might limit our ability to detect other members of global NTM lineages. Third, we may have missed NTM species with specific or fastidious growth requirements, such as *M. marinum*, *M. ulcerans*, *M. haemophilum*, *M. genavense*, and *M. avium* subsp. *paratuberculosis*, which may not have been recoverable under the culture conditions used. Further, our conservative isolation strategy with the use of a single culture medium, temperature and a sample volume of 500 ml might have led to an underestimation of NTM isolation. Nevertheless, we demonstrate the presence of global lineages in household water samples from Germany.

## Conclusion

In this comprehensive study of indoor water samples from Germany, we provide evidence that globally distributed NTM lineages can be detected in local water networks. Further studies should focus on evaluating both the effectiveness of targeted interventions and the potential role of routine environmental sampling alongside clinical surveillance of vulnerable populations. Together, these approaches may provide valuable insights into exposure risks and support the development of more effective prevention strategies.

## Supplementary Information

Supplementary Material 1.

## Data Availability

All sequencing data generated in this study have been uploaded to the European Nucleotide Archive under project number PRJEB88541 (https://www.ncbi.nlm.nih.gov/sra/?term=PRJEB88541). Accession numbers of sequence data generated or used in this study are also available in tables S1-S3.

## References

[1] Griffith DE, Aksamit T, Brown-Elliott BA, Catanzaro A, Daley C, Gordin F, et al. An official ATS/IDSA statement: diagnosis, treatment, and prevention of nontuberculous mycobacterial diseases. Am J Respir Crit Care Med. 2007;175:367–416. 10.1164/rccm.200604-571ST. 17277290 10.1164/rccm.200604-571ST

[2] Runyon EH. Anonymous mycobacteria in pulmonary disease. Med Clin North Am. 1959;43:273–90. 10.1016/S0025-7125(16)34193-1. 13612432 10.1016/s0025-7125(16)34193-1

[3] Daley CL, Iaccarino JM, Lange C, Cambau E, Wallace RJ, Andrejak C, et al. Treatment of nontuberculous mycobacterial pulmonary disease: an official ATS/ERS/ESCMID/IDSA clinical practice guideline. Eur Respir J. 2020. 10.1183/13993003.00535-2020. 32636299 10.1183/13993003.00535-2020 PMC8375621

[4] Marras TK, Mirsaeidi M, Vinnard C, Chan ED, Eagle G, Zhang R, et al. Guidelines-based treatment associated with improved economic outcomes in nontuberculous mycobacterial lung disease. J Med Econ. 2019:1–1. 10.1080/13696998.2019.1620243. 10.1080/13696998.2019.1620243 31094592

[5] Cowman S, van Ingen J, Griffith DE, Loebinger MR. Non-tuberculous mycobacterial pulmonary disease. Eur Respir J. 2019;54:1900250. 10.1183/13993003.00250-2019. 31221809 10.1183/13993003.00250-2019

[6] Hermansen TS, Ravn P, Svensson E, Lillebaek T. Nontuberculous mycobacteria in Denmark, incidence and clinical importance during the last quarter-century. Sci Rep. 2017;7:6696. 10.1038/s41598-017-06931-4. 28751677 10.1038/s41598-017-06931-4 PMC5532240

[7] Dahl VN, Mølhave M, Fløe A, van Ingen J, Schön T, Lillebaek T, et al. Global trends of pulmonary infections with nontuberculous mycobacteria: a systematic review. Int J Infect Dis. 2022. 10.1016/j.ijid.2022.10.013. 36244600 10.1016/j.ijid.2022.10.013

[8] Sommerstein R, Rüegg C, Kohler P, Bloemberg G, Kuster SP, Sax H. Transmission of *Mycobacterium chimaera* from heater-cooler units during cardiac surgery despite an ultraclean air ventilation system. Emerg Infect Dis. 2016;22:1008. 10.3201/EID2206.160045. 27070958 10.3201/eid2206.160045 PMC4880077

[9] Thorel MF, Falkinham JO, Moreau RG. Environmental mycobacteria from alpine and subalpine habitats. FEMS Microbiol Ecol. 2004;49:343–7. 10.1016/j.femsec.2004.04.016. 19712284 10.1016/j.femsec.2004.04.016

[10] Honda JR, Hasan NA, Davidson RM, Williams MD, Epperson LE, Reynolds PR, et al. Environmental nontuberculous mycobacteria in the Hawaiian Islands. PLoS Negl Trop Dis. 2016. 10.1371/JOURNAL.PNTD.0005068. 27780201 10.1371/journal.pntd.0005068 PMC5079566

[11] Wallace RJ, Iakhiaeva E, Williams MD, Brown-Elliott BA, Vasireddy S, Vasireddy R, et al. Absence of *Mycobacterium intracellulare* and presence of *Mycobacterium chimaera* in household water and biofilm samples of patients in the United States with *Mycobacterium avium* complex respiratory disease. J Clin Microbiol. 2013;51:1747. 10.1128/JCM.00186-13. 23536397 10.1128/JCM.00186-13 PMC3716115

[12] Lahiri A, Kneisel J, Kloster I, Kamal E, Lewin A. Abundance of *Mycobacterium avium* ssp. *hominissuis* in soil and dust in Germany - implications for the infection route. Lett Appl Microbiol. 2014;59:65–70. 10.1111/lam.12243. 24612016 10.1111/lam.12243

[13] Honda J, Virdi R, Chan ED. Global environmental nontuberculous mycobacteria and their contemporaneous man-made and natural niches. Front Microbiol. 2018;9:2029. 10.3389/fmicb.2018.02029. 30214436 10.3389/fmicb.2018.02029 PMC6125357

[14] Thomson R, Tolson C, Carter R, Coulter C, Huygens F, Hargreaves M. Isolation of nontuberculous mycobacteria (NTM) from household water and shower aerosols in patients with pulmonary disease caused by NTM. J Clin Microbiol. 2013;51:3006–11. 10.1128/JCM.00899-13. 23843489 10.1128/JCM.00899-13 PMC3754680

[15] Lande L, Alexander DC, Wallace RJ, Kwait R, Iakhiaeva E, Williams M, et al. *Mycobacterium avium* in community and household water, suburban Philadelphia, Pennsylvania, USA, 2010–2012. Emerg Infect Dis. 2019;25:473–81. 10.3201/eid2503.180336. 30789130 10.3201/eid2503.180336 PMC6390762

[16] Song JJX, Oguma K. Mycobacterial contamination in tap and shower waters in Thailand. Lett Appl Microbiol. 2023. 10.1093/LAMBIO/OVAD090. 37528059 10.1093/lambio/ovad090

[17] Peters M, Müller C, Rüsch-Gerdes S, Seidel C, Göbel U, Pohle HD, et al. Isolation of atypical mycobacteria from tap water in hospitals and homes: is this a possible source of disseminated MAC infection in AIDS patients? J Infect. 1995;31:39–44. 10.1016/S0163-4453(95)91333-5. 8522830 10.1016/s0163-4453(95)91333-5

[18] Wetzstein N, AKohl T, Diricks M, Mas-Peiro S, Holubec T, Kessel J, et al. Clinical characteristics and outcome of *Mycobacterium chimaera* infections after cardiac surgery: systematic review and meta-analysis of 180 heater-cooler unit associated cases. Clin Microbiol Infect. 2023. 10.1016/j.cmi.2023.03.005. 36918144 10.1016/j.cmi.2023.03.005

[19] van Ingen J, Kohl TA, Kranzer K, Hasse B, Keller PM, Katarzyna Szafrańska A, et al. Global outbreak of severe *Mycobacterium chimaera* disease after cardiac surgery: a molecular epidemiological study. Lancet Infect Dis. 2017;17:1033–41. 10.1016/S1473-3099(17)30324-9. 28711585 10.1016/S1473-3099(17)30324-9

[20] van Tonder AJ, Ellis HC, Churchward CP, Kumar K, Ramadan N, Benson S, et al. Mycobacterium avium complex (MAC) genomics and transmission in a London hospital. Eur Respir J. 2022;61:2201237. 10.1183/13993003.01237-2022. 10.1183/13993003.01237-2022 PMC10116071 36517182

[21] Painsi C, Lange-Asschenfeldt B. Image gallery: pink papules within a tattoo linked to *Mycobacterium chelonae* infection. Br J Dermatol. 2017;177:e1–e1. 10.1111/bjd.15481. 28731256 10.1111/bjd.15481

[22] Padilla P, Ly P, Dillard R, Boukovalas S, Zapata-Sirvent R, Phillips LG. Medical tourism and postoperative infections: a systematic literature review of causative organisms and empiric treatment. Plast Reconstr Surg. 2018;142:1644–51. 10.1097/PRS.0000000000005014. 30489537 10.1097/PRS.0000000000005014

[23] Kikhney J, Friesen I, Wiesener S, Kursawe L, Loddenkemper C, Zündorf J, et al. Endocarditis associated with contamination of cardiovascular bioprostheses with *Mycobacterium **chelonae*: a collaborative microbiological study. Lancet Microbe. 2024;0:100934. 10.1016/j.lanmic.2024.06.001. 10.1016/j.lanmic.2024.06.001 39491876

[24] Wetzstein N, Diricks M, Kohl TA, Wichelhaus TA, Andres S, Paulowski L, et al. Molecular epidemiology of mycobacterium abscessus isolates recovered from german cystic fibrosis patients. Microbiol Spectr. 2022:e0171422. 10.1128/SPECTRUM.01714-22. 10.1128/spectrum.01714-22 PMC9431180 35938728

[25] Diricks M, Merker M, Wetzstein N, Kohl TA, Niemann S, Maurer FP. Delineating *Mycobacterium abscessus* population structure and transmission employing high-resolution core genome multilocus sequence typing. Nat Commun. 2022;13:1–10. 10.1038/s41467-022-32122-5. 35999208 10.1038/s41467-022-32122-5 PMC9399081

[26] Bryant JM, Brown KP, Burbaud S, Everall I, Belardinelli JM, Rodriguez-Rincon D, et al. Stepwise pathogenic evolution of *Mycobacterium abscessus*. Science. 2021. 10.1126/science.abb8699. 33926925 10.1126/science.abb8699 PMC7611193

[27] Thomson RM, Wheeler N, Stockwell RE, Bryant J, Taylor SL, Leong LEX, et al. Infection by clonally related *Mycobacterium abscessus* isolates: the role of drinking water. Am J Respir Crit Care Med. 2025. 10.1164/rccm.202409-1824OC. 40072241 10.1164/rccm.202409-1824OC PMC12091025

[28] European Centre for Disease Prevention and Control. EU protocol for case detection, laboratory diagnosis and environmental testing of Mycobacterium chimaera infections potentially associated with heater-cooler units: case definition and environmental testing methodology. Stockholm: ECDC; 2015. https://www.ecdc.europa.eu/en/publications-data/eu-protocol-case-detection-laboratory-diagnosis-and-environmental-testing.

[29] Richter E, Beer J, Diel R, et al. Qualitätsstandards in der mikrobiologisch-infektiologischen diagnostik: MIQ--5 tuberkulose mybakteriose. Elsevier, Urban und Fischer, München; 2010. ISBN-13: 978-3437415319

[30] Woods GL, Wengenack NL, Grace Lin D, Barbara Brown-Elliott MA, Daniela Maria Cirillo M, Conville PS, et al. M62: performance standards for susceptibility testing of mycobacteria, nocardia spp., and other aerobic actinomycetes. 2018. Available: www.clsi.org. 31339680

[31] de Almeida IN, Carvalho WDS, Rossetti ML, Costa ERD, de Miranda SS. Evaluation of six different DNA extraction methods for detection of *Mycobacterium tuberculosis* by means of PCR-IS6110: preliminary study. BMC Res Notes. 2013;6:561. 10.1186/1756-0500-6-561. 24373461 10.1186/1756-0500-6-561 PMC3891981

[32] Baym M, Kryazhimskiy S, Lieberman TD, Chung H, Desai MM, Kishony R. Inexpensive multiplexed library preparation for megabase-sized genomes. PLoS One. 2015;10:e0128036. 10.1371/journal.pone.0128036. 26000737 10.1371/journal.pone.0128036 PMC4441430

[33] NTMtools/scripts/NTMseq at main · ngs-fzb/NTMtools. Available: https://github.com/ngs-fzb/NTMtools/tree/main/scripts/NTMseq. Cited 28 May 2024.

[34] GitHub - s-andrews/FastQC: a quality control analysis tool for high throughput sequencing data. Available: https://github.com/s-andrews/FastQC. Cited 11 Aug 2023.

[35] Ewels P, Magnusson M, Lundin S, Käller M. Multiqc: summarize analysis results for multiple tools and samples in a single report. Bioinformatics. 2016;32:3047–8. 10.1093/bioinformatics/btw354. 27312411 10.1093/bioinformatics/btw354 PMC5039924

[36] Wood DE, Lu J, Langmead B. Improved metagenomic analysis with Kraken 2. Genome Biol. 2019;20:257. 10.1186/s13059-019-1891-0. 31779668 10.1186/s13059-019-1891-0 PMC6883579

[37] GitHub - jodyphelan/NTM-profiler: profiling NTM WGS data. Available: https://github.com/jodyphelan/NTM-Profiler. Cited 11 Aug 2023.

[38] GitHub - tseemann/shovill: assemble bacterial isolate genomes from Illumina paired-end reads. Available: https://github.com/tseemann/shovill. Cited 11 Aug 2023.

[39] Bankevich A, Nurk S, Antipov D, Gurevich AA, Dvorkin M, Kulikov AS, et al. SPAdes: a new genome assembly algorithm and its applications to single-cell sequencing. J Comput Biol. 2012;19:455–77. 10.1089/cmb.2012.0021. 22506599 10.1089/cmb.2012.0021 PMC3342519

[40] Feldgarden M, Brover V, Gonzalez-Escalona N, Frye JG, Haendiges J, Haft DH, et al. AMRFinderPlus and the reference gene catalog facilitate examination of the genomic links among antimicrobial resistance, stress response, and virulence. Sci Rep. 2021;11:12728. 10.1038/s41598-021-91456-0. 34135355 10.1038/s41598-021-91456-0 PMC8208984

[41] Inouye M, Dashnow H, Raven LA, Schultz MB, Pope BJ, Tomita T, et al. SRST2: rapid genomic surveillance for public health and hospital microbiology labs. Genome Med. 2014;6:90. 10.1186/s13073-014-0090-6. 25422674 10.1186/s13073-014-0090-6 PMC4237778

[42] Meier-Kolthoff JP, Auch AF, Klenk HP, Göker M. Genome sequence-based species delimitation with confidence intervals and improved distance functions. BMC Bioinformatics. 2013. 10.1186/1471-2105-14-60. 23432962 10.1186/1471-2105-14-60 PMC3665452

[43] Lefort V, Desper R, Gascuel O. FastME 2.0: a comprehensive, accurate, and fast distance-based phylogeny inference program: table 1. Mol Biol Evol. 2015;32:2798–800. 10.1093/molbev/msv150. 26130081 10.1093/molbev/msv150 PMC4576710

[44] Meier-Kolthoff JP, Carbasse JS, Peinado-Olarte RL, Göker M. TYGS and LPSN: a database tandem for fast and reliable genome-based classification and nomenclature of prokaryotes. Nucleic Acids Res. 2022;50:D801-7. 10.1093/nar/gkab902. 34634793 10.1093/nar/gkab902 PMC8728197

[45] Letunic I, Bork P. Interactive tree of life (iTOL) v5: an online tool for phylogenetic tree display and annotation. Nucleic Acids Res. 2021;49:W293-6. 10.1093/nar/gkab301. 33885785 10.1093/nar/gkab301 PMC8265157

[46] Yu G, Smith D, Zhu H, Guan Y, Lam TTY. Ggtree: an R package for visualization and annotation of phylogenetic trees with their covariates and other associated data. Methods Ecol Evol. 2017;8:28–36. 10.1111/2041-210X.12628.

[47] R Core Team. R: a language and environment for statistical computing. Vienna; 2018. Available: https://www.r-project.org/.

[48] Wickham H. ggplot2: elegant graphics for data analysis. Springer-Verlag New York; 2016. Available: https://ggplot2.tidyverse.org.

[49] Kahle D, Wickham H. ggmap: spatial visualization with ggplot2. R J. 2013;5:144–161. Available: https://journal.r-project.org/archive/2013-1/kahle-wickham.pdf.

[50] Falkinham JO III. Nontuberculous mycobacteria from household plumbing of patients with nontuberculous mycobacteria disease. Emerg Infect Dis. 2011;17:419–24. 10.3201/eid1703.101510. 21392432 10.3201/eid1703.101510 PMC3166028

[51] Ichijo T, Izumi Y, Nakamoto S, Yamaguchi N, Nasu M. Distribution and respiratory activity of mycobacteria in household water system of healthy volunteers in Japan. PLoS ONE. 2014;9:e110554. 10.1371/journal.pone.0110554. 25350137 10.1371/journal.pone.0110554 PMC4211706

[52] Tzou CL, Dirac MA, Becker AL, Beck NK, Weigel KM, Meschke JS, et al. Association between *mycobacterium avium* complex pulmonary disease and mycobacteria in home water and soil a case-control study. Ann Am Thorac Soc. 2020;17:57–62. 10.1513/AnnalsATS.201812-915OC. 31644315 10.1513/AnnalsATS.201812-915OC PMC6944351

[53] van Ingen J, Blaak H, de Beer J, de Roda Husman AM, van Soolingen D. Rapidly growing nontuberculous mycobacteria cultured from home tap and shower water. Appl Environ Microbiol. 2010;76:6017–9. 10.1128/AEM.00843-10. 20639378 10.1128/AEM.00843-10 PMC2935072

[54] Kobayashi M, Oana K, Kawakami Y. Bath water contamination with *Legionella* and nontuberculous mycobacteria in 24-hour home baths, hot springs, and public bathhouses of Nagano prefecture, Japan. Jpn J Infect Dis. 2014;67:276–81. 10.7883/yoken.67.276. 25056073 10.7883/yoken.67.276

[55] Shi X, Wang J, Huang X, Sha W, Qin L. Isolation of non-tuberculous mycobacteria (NTM) from household water of patients with NTM pulmonary disease. Altern Ther Health Med. 2024;30:79–83. 38581318

[56] Monde N, Munyeme M, Muwonge A, Muma JB, Malama S. Characterization of non-tuberculous mycobacterium from humans and water in an agropastoral area in Zambia. BMC Infect Dis. 2018. 10.1186/s12879-017-2939-y. 29310592 10.1186/s12879-017-2939-y PMC5759224

[57] Moghaddam S, Nojoomi F, Dabbagh Moghaddam A, Mohammadimehr M, Sakhaee F, Masoumi M, et al. Isolation of nontuberculous mycobacteria species from different water sources: a study of six hospitals in Tehran, Iran. BMC Microbiol. 2022. 10.1186/s12866-022-02674-z. 36309645 10.1186/s12866-022-02674-z PMC9617398

[58] Wetzstein N, Kohl TA, Andres S, Schultze TG, Geil A, Kim E, et al. Comparative analysis of phenotypic and genotypic antibiotic susceptibility patterns in *Mycobacterium avium* complex. Int J Infect Dis. 2020;93:320–8. 10.1016/j.ijid.2020.02.059. 32147539 10.1016/j.ijid.2020.02.059

[59] Wetzstein N, Diricks M, Anton TB, Andres S, Kuhns M, Kohl TA, et al. Clinical and genomic features of *Mycobacterium avium* complex: a multi-national European study. Genome Med. 2024;16:86. 10.1186/s13073-024-01359-8. 38982539 10.1186/s13073-024-01359-8 PMC11232273

[60] VandeWeygaerde Y, Cardinaels N, Bomans P, Chin T, Boelens J, André E, et al. Clinical relevance of pulmonary non-tuberculous mycobacterial isolates in three reference centres in Belgium: a multicentre retrospective analysis. BMC Infect Dis. 2019;19:1–10. 10.1186/S12879-019-4683-Y. 31847834 10.1186/s12879-019-4683-y PMC6918577

[61] Walker TM, Ip CLC, Harrell RH, Evans JT, Kapatai G, Dedicoat MJ, et al. Whole-genome sequencing to delineate *Mycobacterium tuberculosis* outbreaks: a retrospective observational study. Lancet Infect Dis. 2013;13:137–46. 10.1016/S1473-3099(12)70277-3. 23158499 10.1016/S1473-3099(12)70277-3 PMC3556524

[62] Fine PEM, Crampin AC, Houben RMGJ, Mzembe T, Mallard K, Coll F, et al. Large-scale whole genome sequencing of M. tuberculosis provides insights into transmission in a high prevalence area. Elife. 2015. 10.7554/eLife.05166. 25732036 10.7554/eLife.05166 PMC4384740

