# Supplementary material for: Global lineages of non-tuberculous mycobacteria in residential water samples from Germany
Source: BMC Microbiol. 2025 Dec 7;25:792. doi: 10.1186/s12866-025-04563-7 (PMC12701587; doi:10.1186/s12866-025-04563-7)
Supplement: Supplementary file 1 — Supplementary Material 1. [file 12866_2025_4563_MOESM1_ESM.zip › supplementals_20251016.docx]

**Supplementary Tables**

Table S1: Accession codes and metadata of all sequence data used in this study for *M. chimaera*.

Table S2: Accession codes and metadata of all sequence data used in this study for *M. chelonae*.

Table S3: Accession codes and metadata of all sequence data used in this study for *M. abscessus*.

Table S4: List of genes used in the cgMLST schemes for *M. chimaera* and *M. chelonae*

Table S5: SLOMYCO1 MICs of environmental SGM recovered in this study.

Table S6: RAMPYCO1 MICs of environmental RGM samples recovered in this study.

**Supplementary Figures**

Figure S1: Exemplary images of faucets (A-C) and shower heads (D-F) from which water samples were retrieved.


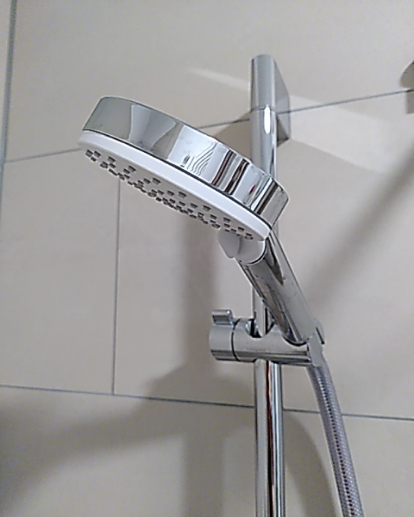

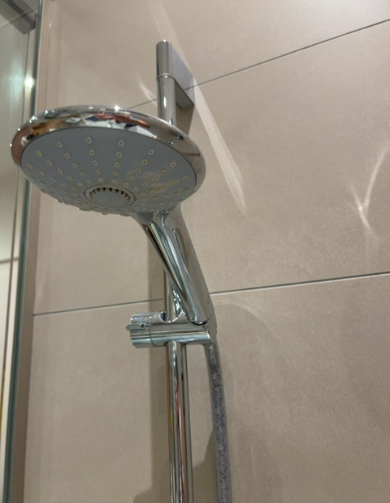

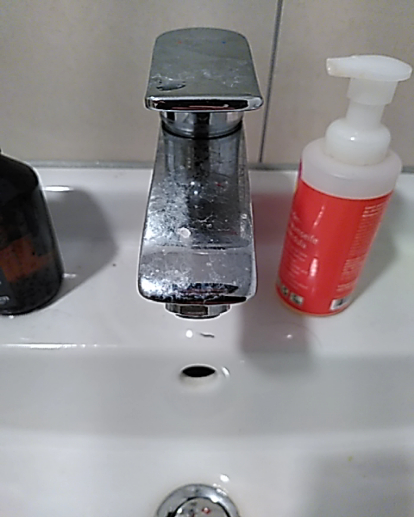

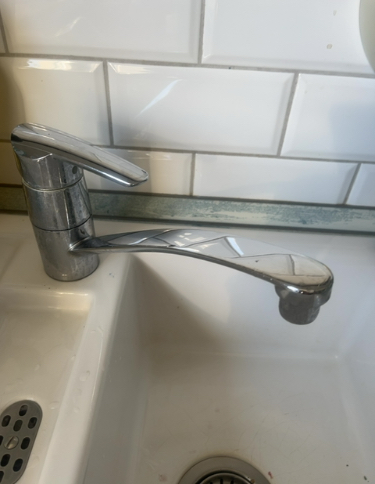

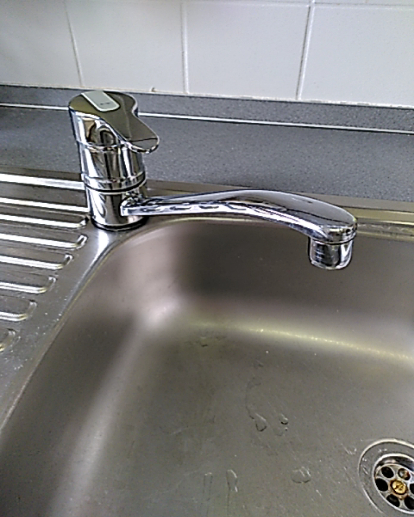

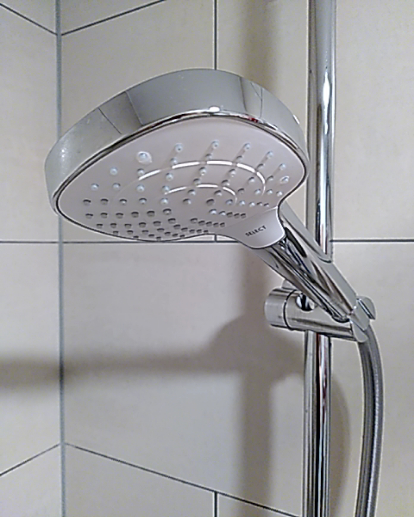


A

B

C

F

E

D


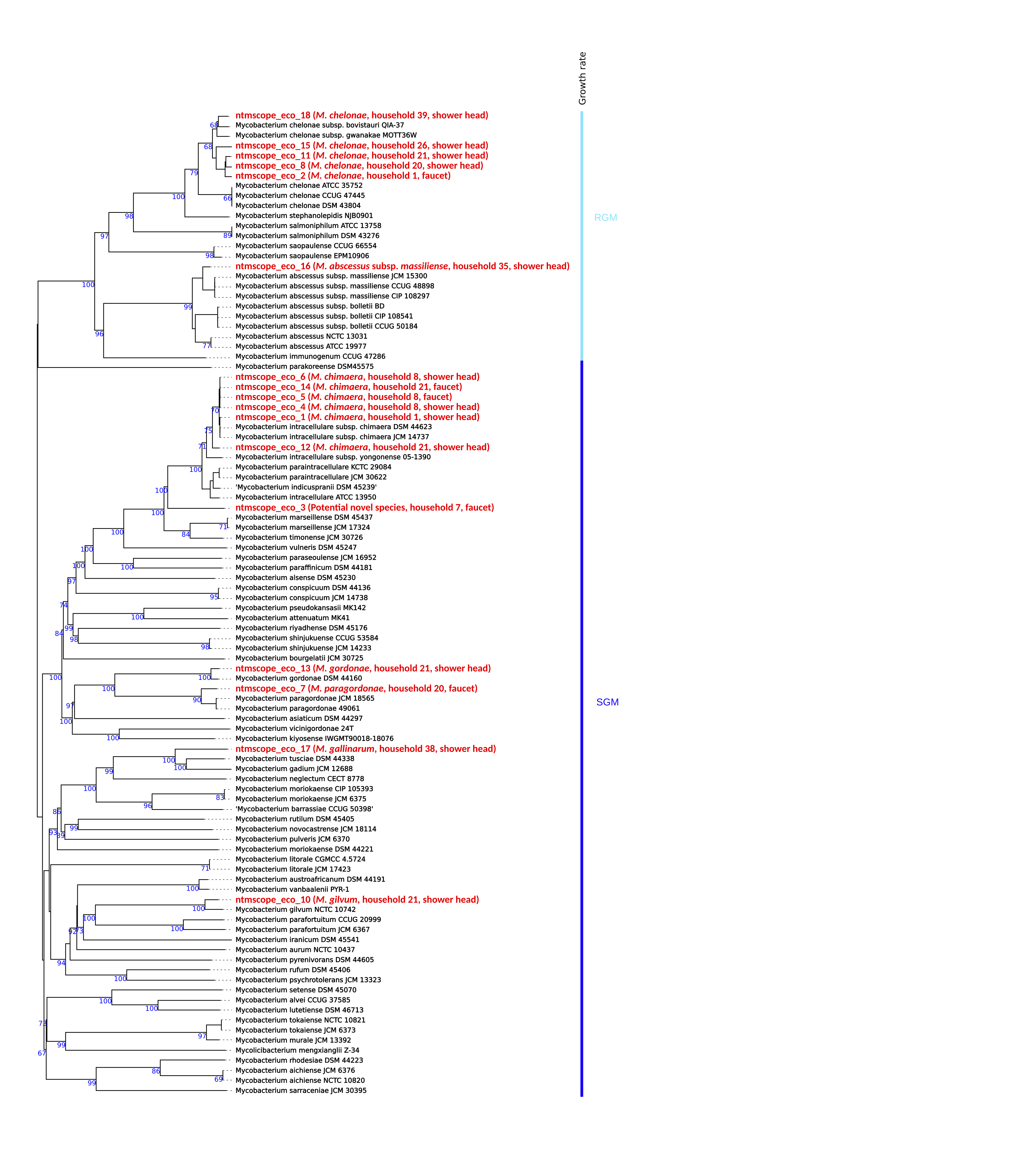


Figure S2: Overall phylogeny of recovered NTM isolates and comparative reference genomes.


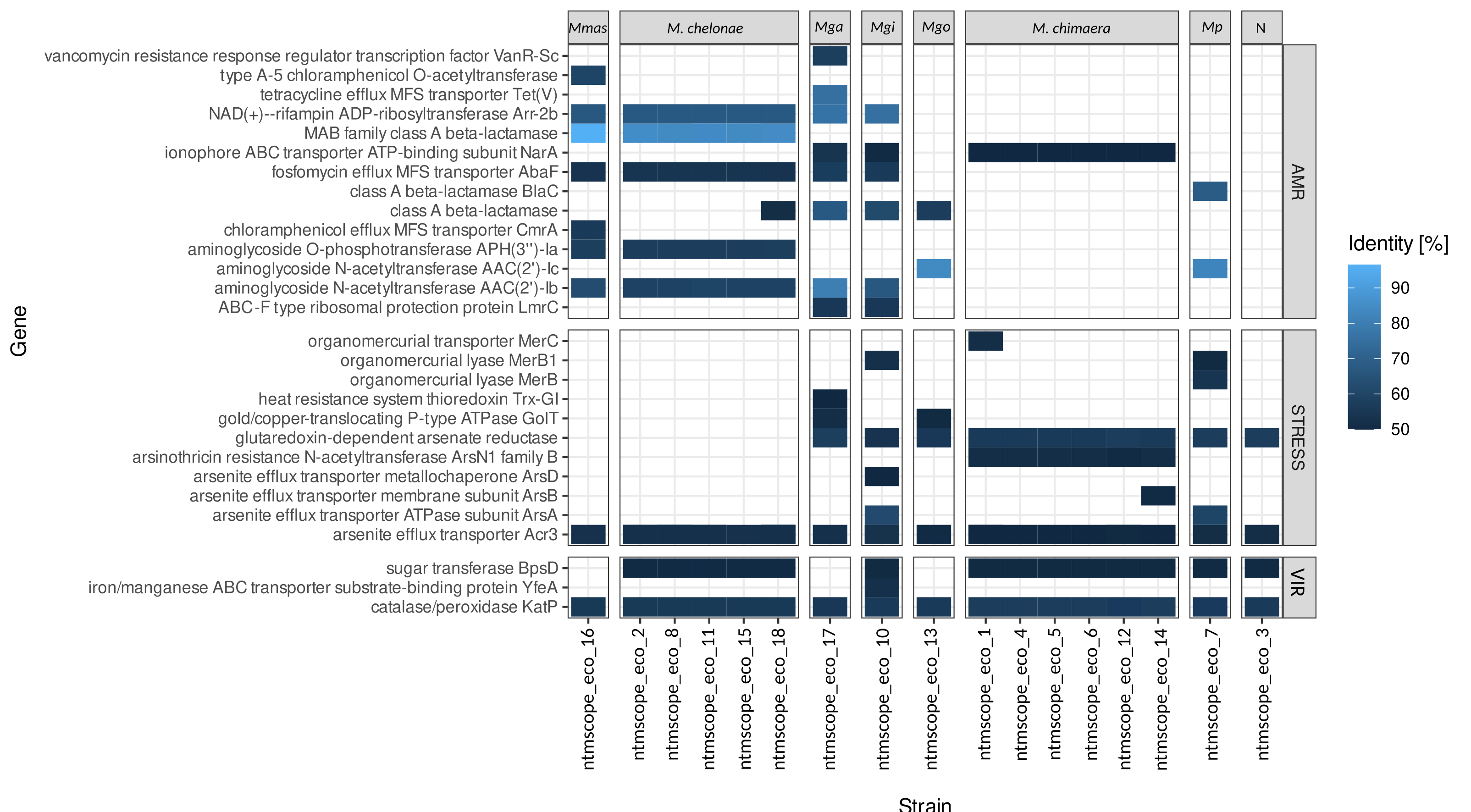


Figure S3: Detection of antimicrobial resistance, stress and virulence genes in recovered NTM isolates. *Mmas – M. abscessus* subsp. *massiliense*; *Mga – M. gallinarum; Mgi – M. gilvum; Mgo – M. gordonae; Mp – M. paragordonae*; N – Novel species; VIR – virulence.


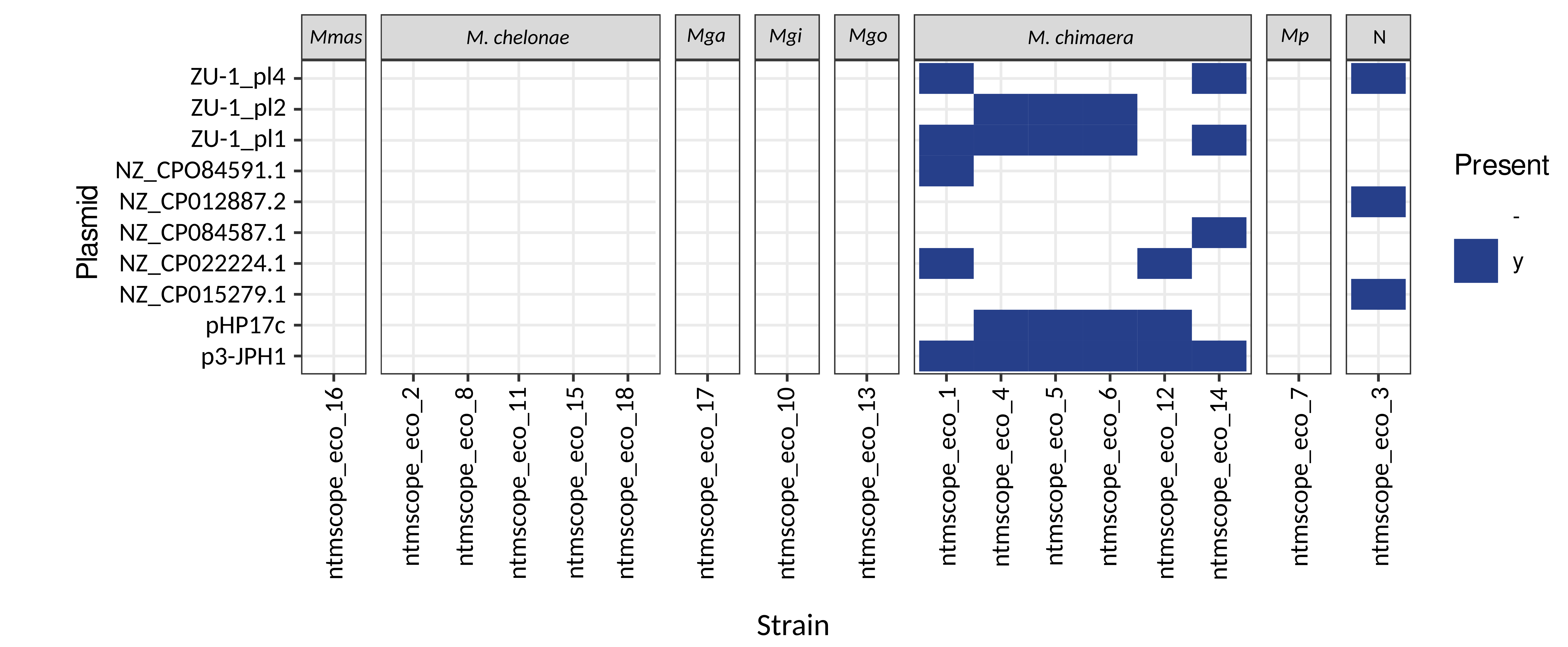
Figure S4: Predicted plasmids in recovered environmental NTM isolates. Graph shows only plasmids that were detected in at least one environmental NTM isolate. *Mmas – M. abscessus* subsp. *massiliense*; *Mga – M. gallinarum; Mgi – M. gilvum; Mgo – M. gordonae; Mp – M. paragordonae*; N – Novel species; VIR – virulence.


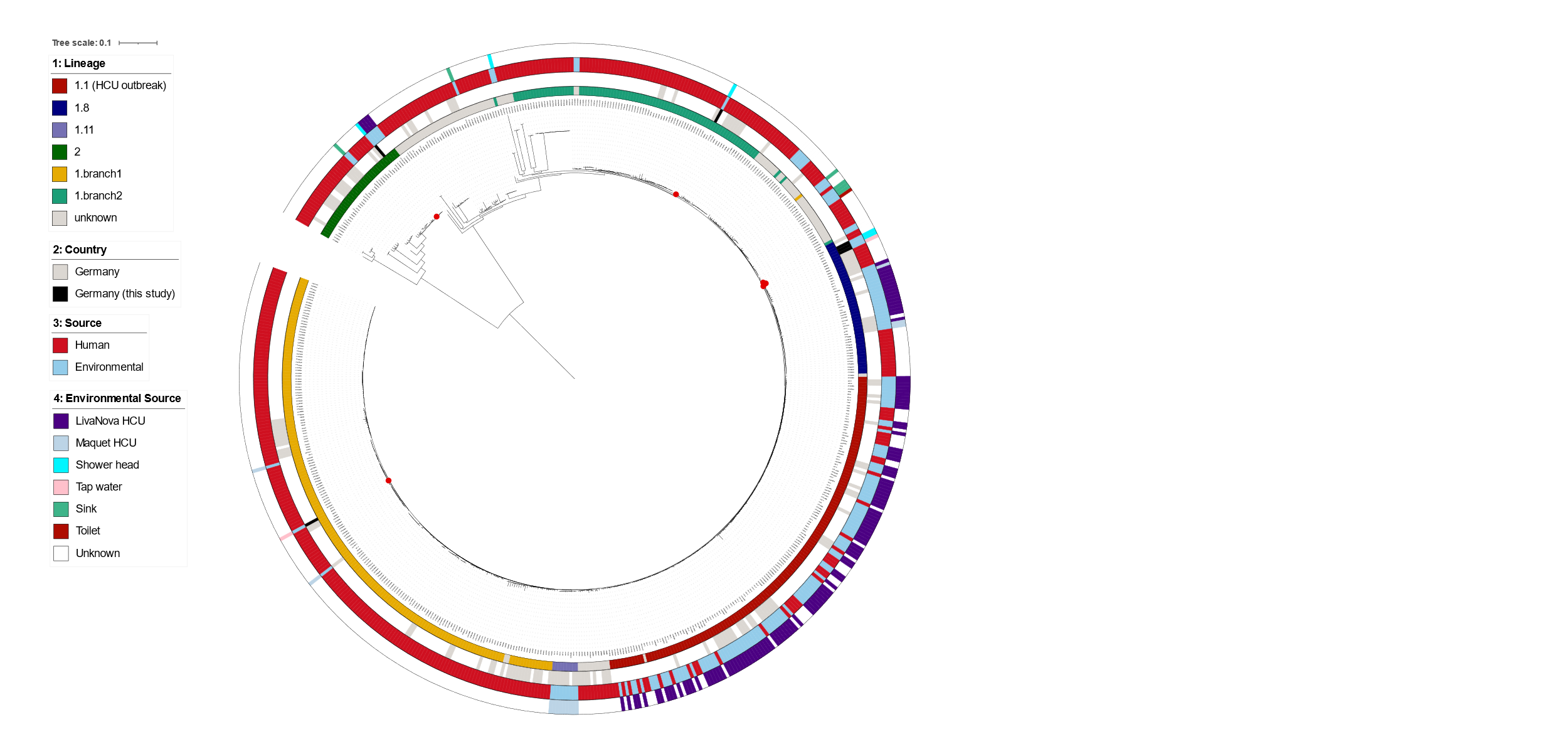


Figure S5: Phylogeny of 6 *M. chimaera* isolates recovered from environmental samples in this study (red dots) and 607 public sequences. Neighbor joining tree was constructed using SeqSphere+ based on 3719 cgMLST targets and annotated using iTOL.


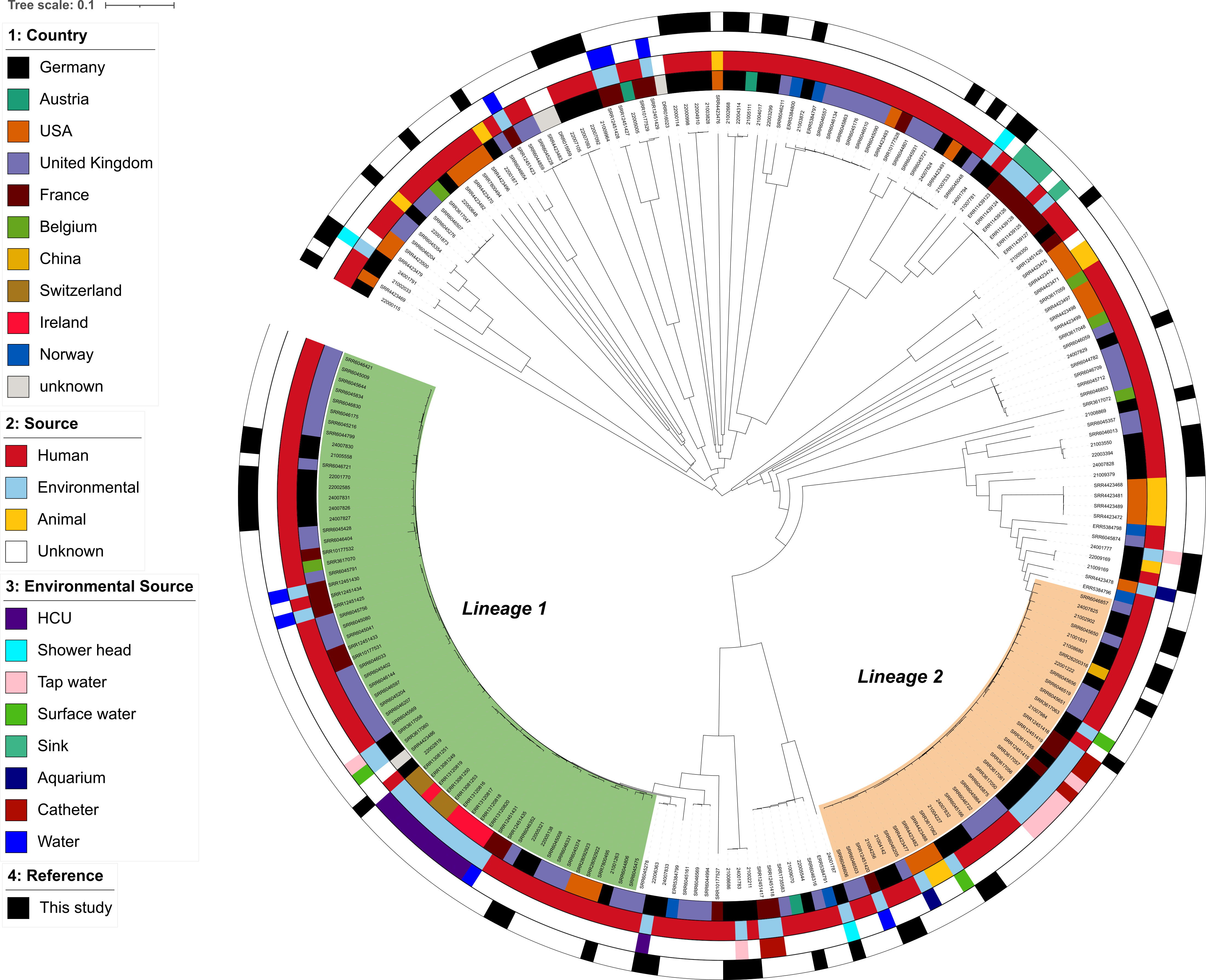


Figure S6: Phylogeny of 225 *M. chelonae* isolates. Ntmscope_eco_9 was excluded due to contamination with proteobacteria. The two main lineages (lineage 1: d50 cluster 1 and lineage 2: d50 cluster 2) defined with a core genome multilocus sequence typing (cgMLST) distance threshold of 50 alleles are visualised in green and orange. Neighbor joining tree was constructed using SeqSphere+ based on 3461 cgMLST targets and annotated using iTOL.


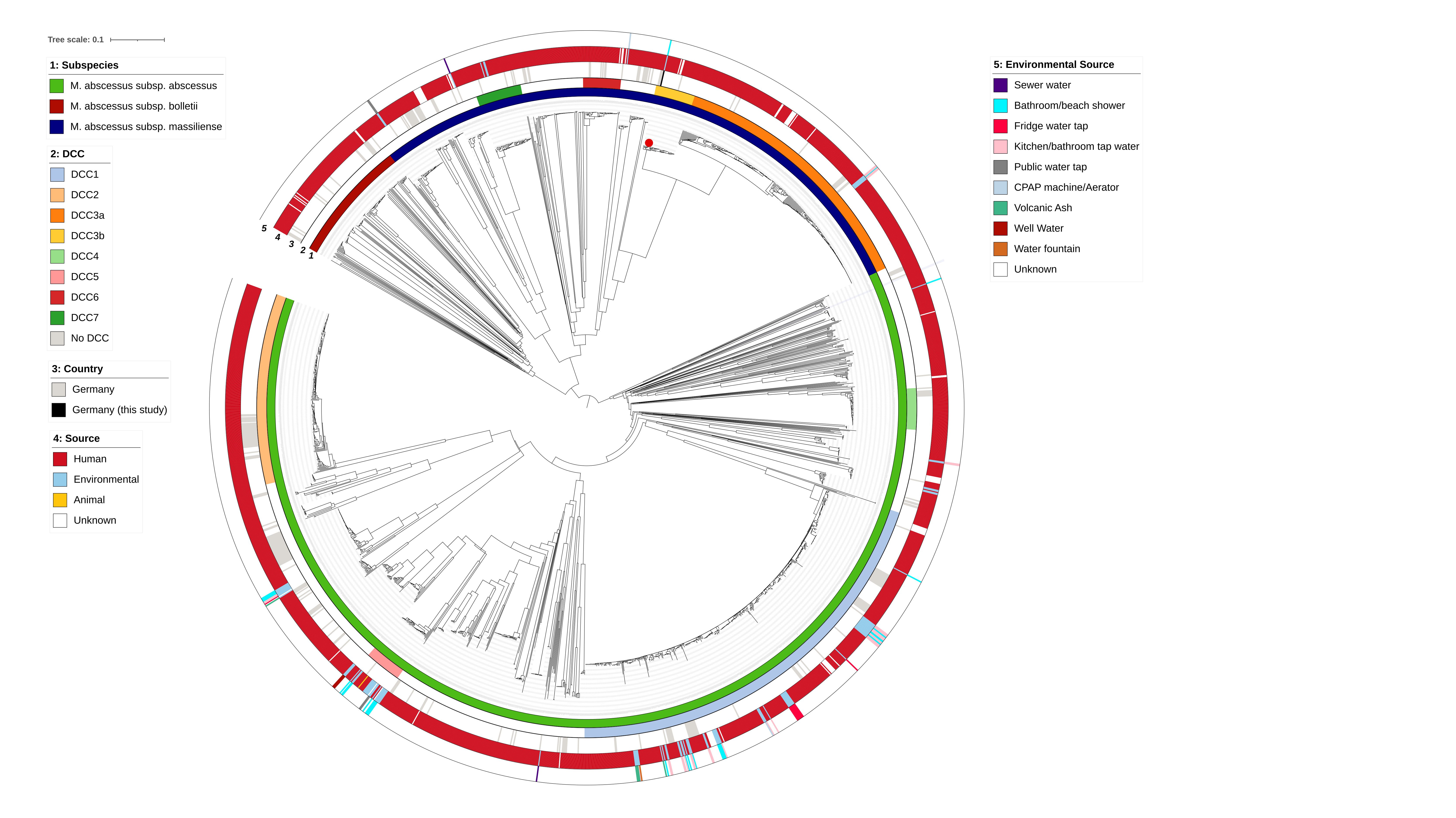
Figure S7: Phylogeny of one *M. abscessus* subsp. *massiliense* isolate recovered from an indoor water sample in this study (red dot) belonging to DCC3b and 2131 public sequences. Neighbor joining tree was constructed using SeqSphere+ based on 2904 cgMLST targets and annotated using iTOL.
